# Cloud-enabled e-commerce negotiation framework using bayesian-based adaptive probabilistic trust management model

**DOI:** 10.1038/s41598-025-92643-z

**Published:** 2025-03-19

**Authors:** Rajkumar Rajavel, Lalitha Krishnasamy, Partheeban Nagappan, Usha Moorthy, Sathishkumar Veerappampalayam Easwaramoorthy

**Affiliations:** 1 Department of Computer Science and Engineering, Christ University, Bengaluru, 560074 India; 2 Department of Artificial Intelligence and Data Science, Nandha Engineering College, Erode, Tamil Nadu India; 3 Department of Computer Science and Engineering, Galgotias University, Greater Noida, India; 4 Department of Information Technology, Manipal Institute of Technology Bengaluru, Manipal Academy of Higher Education, Manipal, India; 5 Department of Data Science and Artificial Intelligence, Sunway University, No. 5, Jalan Universiti, Bandar Sunway, Selangor Darul Ehsan, Subang Jaya, 47500 Malaysia

**Keywords:** Cloud computing, Multi-agent system, Broker-based negotiation, E-commerce negotiation framework, Bayesian learning, Adaptive probabilistic trust management model, Engineering, Mathematics and computing

## Abstract

Enforcing a trust management model in the broker-based negotiation context is identified as a foremost challenge. Creating such trust model is not a pure technical issue, but the technology should enhance the cloud service negotiation framework for improving the utility value and success rate between the bargaining participants (consumer, broker, and service provider) during their negotiation progression. In the existing negotiation frameworks, trusts were established using reputation, self-assessment, identity, evidence, and policy-based evaluation techniques for maximizing the negotiators (cloud participants) utility value and success rate. To further maximization, a Bayesian-based adaptive probabilistic trust management model is enforced in the future broker-based trusted cloud service negotiation framework. This adaptive model dynamically ranks the service provider agents by estimating the success rate, cooperation rate and honesty rate factors to effectively measure the trustworthiness among the participants. The measured trustworthiness value will be used by the broker agents for prioritization of trusted provider agents over the non-trusted provider agents which minimizes the bargaining conflict between the participants and enhance future bargaining progression. In addition, the proposed adaptive probabilistic trust management model formulates the sequence of bilateral negotiation process among the participants as a Bayesian learning process. Finally, the performance of the projected cloud-enabled e-commerce negotiation framework with Bayesian-based adaptive probabilistic trust management model is compared with the existing frameworks by validating under different levels of negotiation rounds.

## Introduction

Establishing a negotiation framework is one of the major challenging issues in the Service Level Agreement (SLA)-oriented cloud management system for supporting the customized resource provisioning mechanism in cloud market^1,2^. In a negotiation framework, the negotiating participants follow different communication patterns like one-to-one, one-to-many, many-to-many with flooding and many-to-many with ring traversal during their bilateral negotiation process^3^. Among these patterns, many-to-many communication pattern is an appropriate model for realizing the situations of many real-world negotiation problems. Therefore, most of the researchers suggest this pattern for emerging research explorations and for application development in complex negotiation problems. In general, negotiation is the type of decision-making approach used to reach an agreement in the sense of psychological, social, and economic aspects among whenever a person or an organization entity cannot achieve its objectives and goals unilaterally^4^. In a broader sense negotiation is defined as the process of planning, exploring, trading, deciding, and implementing in situation such as converging and opposing interests^5^. Further, the emerging concept of e-negotiation creates the opportunities for the e-commerce researchers and practitioners to negotiate with business partners located at remote place, at any time. Here, the participants of e-negotiations may be human entities or intelligent software agent entities. According to the necessity and scope, the degree of humans and software agents’ involvement were applied in mixed type of e-negotiations, in which human entities make higher level decisions while software agent entities are involved in computational estimations^6^. In addition, the software agents are used for automating the negotiation process on behalf of their participants since they significantly reduce the negotiation time and can also remove the reticence of human entity to engage in negotiation process^7^.

Automated negotiation process involves the strategy of higher-level bargaining communication protocol and negotiation strategy for agent interaction. The bargaining protocol expresses the rule of meeting between the negotiating members under which the sequence of offers is exchanged between the interacting agents. On the other hand, an agent’s negotiation strategy denotes the specification of negotiation scenario that makes the sequence of decisions and actions followed during the generation of agent’s negotiation offer^8^. In a complex multi-agent negotiation system due to lack of time and resource, it is not possible for each negotiator available at one part to negotiate with all the potential counterparts. In this connection, the presence of past negotiation experiences can be helpful to judge the value of counterparts for assigning preferences in future negotiations. Therefore, an appropriate negotiation counterpart’s decision is needed to select the right counterparts and reject the wrong ones which have a significant negative impact on the negotiation success rate^9^. Also to further maximize the success rate, an intelligent time-oriented, resource-oriented, and behavior-oriented negotiation strategy (tactics) is exploited in the existing negotiation framework to effectively select an appropriate counterpart^10^.

One of the key components required for the negotiation relationship establishment is trust. The problem of enforcing trust in negotiations is tends to view the trust relationships as the complex, multi-faceted, and changing over time. This problem inspires this research study towards the establishment of trusted negotiation framework model in the multi-cloud infrastructure. Therefore, the key objective of the proposed research study is to develop the cloud-enabled e-commerce negotiation framework (CENF) for improving the utility value and success rate between the negotiating participants (consumer, broker, and provider). The most important contribution of this research work includes: (1) an architecture of broker-based cloud-enabled e-commerce negotiation framework using agent-based technology and alternate offer protocol mechanism that can effectively manage the negotiation cooperation among the participants, (2) a novel Bayesian-based Adaptive Probabilistic Trust Management Model to suggest the best and trustable service provider for the consumer, based on past negotiation offers, (3) incorporate a peer-to-peer trust representation, based on degree of success rate, cooperation rate, and honesty rate to analyze the opponent offer. The proposed trust management model in the negotiation framework will be appropriate to solve several real-world e-commerce problems such as online purchase, automatic flight controller and stock market business.

The rest of the article is prepared as pursued. Subsequent section proposed with literature reviews demonstrating the concise start-of-the-art information about the trust management model and agent-based negotiation framework in a cloud environment. In problem formulation section, the origins of research problem related to negotiation framework and trust evaluation metrics are stated with objective function. Further section describes the effective modeling of Bayesian-based Adaptive Probabilistic Trust Management model. Cloud-enabled e-commerce negotiation framework section deals with the architectural design of both negotiation framework and its adaptive trust management model. Experimental evaluation part finally demonstrates the real time negotiation activity carried out in cloud service e-commerce application using famous JADE toolkit with performance evaluation graph.

## Literature reviews

The emotions play a major role in the development of negotiation relationship among the participants and it influences the concept of negotiation process and its related interactions. These emotional expressions in the negotiators can increases the confidence in their judgment, problem solving, favorable outcomes (success rate), and offer concession^11^. As per the current state-of-the-art research studies, the role of emotional expressions like happiness and anger has been neglected that emphasizes the negotiation strategy and information processing. In real world negotiations, problems occur in the context of existing relationship between the negotiating parties where the past and future play a major role. After establishing the relationship between the negotiators, the corresponding dynamics of the bargaining process are altered; negotiating parties alter their strategies, tactics, and objectives^12^.

Enforcing trust models in the cloud environment are beneficial to cloud users, cloud providers, clouds-of-clouds collaborators, and external auditors^13^. More recently, broker (agency) based cloud resource provisioning is identified as an emerging research area in cloud environment^14–18^. The utility value can be maximized by optimizing the negotiation strategies (rules and policies) of the cloud service negotiating participants^19^. These negotiation strategies could be optimized in the context of pre-request or long-term bargaining process^20^. In addition, the enforcement of both trust-based negotiation framework and strategy in the projected research study can optimize the success rate between the bargaining participants. Many cloud computing literatures are separately available in the aspects of broker-based negotiation framework^21–24^ and broker-based trust management model^25–28^. None of the researchers have focused their research in the combined context of broker-based trusted negotiation framework which tightly integrates the concept of negotiation with the trust model. Trust model is the integral part of negotiation and relationship management. In this concern, different dimension of bargaining relationship, influence, and trust will disturb the contribution of negotiator’s outcome^29^. So, trust can be established both as an antecedent and a consequence in relationship development process. To effectively develop the trust model in the negotiation framework, researchers want to understand the multifaceted landscape of trust model and its progressive dynamics. So, cloud-based trusted negotiation literatures are organized into two sections, namely trust management models and trusted negotiation frameworks.

### Trust management models

**Fig. 1:**
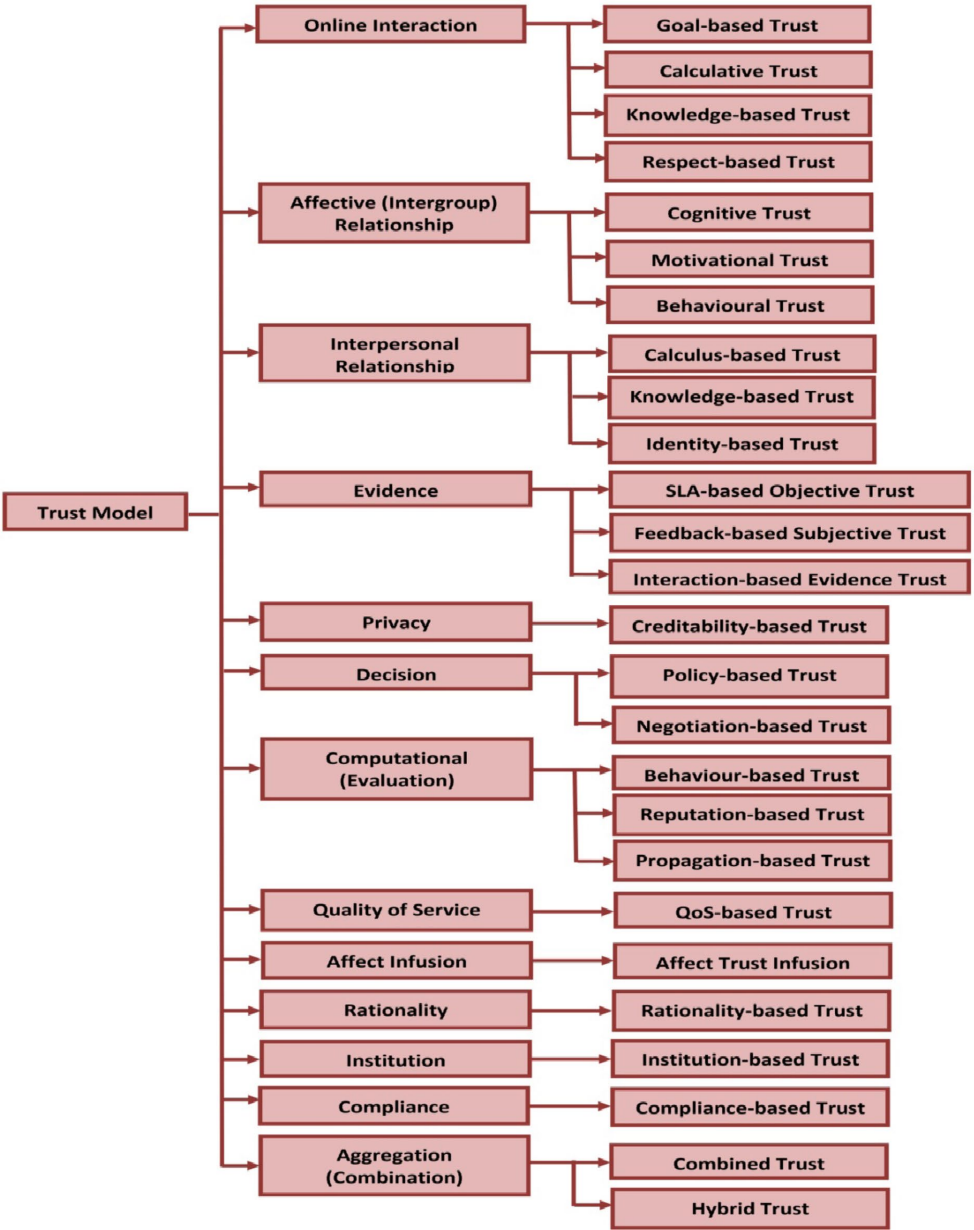
Taxonomy of cloud-based Trust Model.

In general, cloud-enabled trust management model could be broadly analyzed according to the context of online interaction^30^, affective (intergroup) relationship^31^, interpersonal relationship^32,33^, evidence^34,35^, privacy^36–38^, decision, evaluation (computational)^39,40^, quality of service^41^, affect infusion^42^, rationality^43^, institution^44^, compliance^45^, and aggregation (combination)^46–48^. The different trust models associated with these classifications are represented in the classification of trust management models as exposed in Fig. 1. A development of agent-based trust management model is intensively pragmatic and dependent upon circumstances like landscape of the parties, transaction, connection among the parties, and credibility of external forces^49^. Now-a-days, so many service providers available in cloud market to sell their services at varying prices and quality. So, a trust-based management model that quantifies the quality of service provided by the various providers and assist the consumers to select the appropriate cloud provider^50^. Therefore, several trust management models based on various trust evaluation metrics have been analyzed from the literature to develop an adaptive trust model which can satisfies the requirement of consumers in the cloud environment. In the context of enhancing privacy preservation capabilities, the federated recommender systems have gained more attention in the recent years to achieve collaborative learning in the real-world scenarios that can defend against the malicious users and poisoning attacks^51,52^. As per the latest survey on trust management model, more comprehensive analysis is done in the context of Bayesian approach with more involvement of probability theory. One of the best possible ways to fill the trust management gap and analyze the intrinsic trust model relationship through the usage of generic Bayesian trust modeling and evaluation metrics^53^. Still, there are some possible vulnerable attacks like energy depletion and data pollution were observed in the edge-cloud based collaborative networking environment. So, a novel ensemble learning-based trust management mechanism is explored in the edge and cloud servers by collecting trust evidences from all the cluster nodes^54^. In the intelligent transportation scenarios, a novel blockchain-based vehicle reputation model is proposed to suppress the malicious and selfish behaviors according to the estimation of reputation deposit presented over smart contracts^55^.

In the context of cloud-based trust management models, some of the recent research studies were identified in the vehicular network environment that can highlights the various forms of trust evaluation models. A context-awareness trust assessment model is explored with Reinforcement Learning techniques to measure the trustworthiness of messages exchanges happening among the vehicles used in the vehicular network^56^. Similarly, a trust management system based on the cascading-based emergency message dissemination model is exploited with data-oriented trust evaluation approach to improve the robustness among the vehicles and overcome the malicious behaviors and attacks^57^. To ensure the privacy-preservation among the cloud-based vehicular networks, a novel privacy-preserving reputation updating mechanism has been adapted with elliptic curve cryptography approach to minimize the computation and communication overhead^58^. Similarly, a privacy-preserving trust management scheme has been introduced for real-time message dissemination in the space–air–ground embedded networking environment with more robustness and less communication overhead^59^. These types of privacy-preservation coarse-grained and simplex trust evaluation mechanisms may not be the best fit model for the federated learning environment that has varying user behavior patterns^60^. Now-a-days both the trust management and privacy-preservation play a key role in the vehicular networks and there exist the tradeoff between both the schemes. Therefore, a novel hybrid scheme has been introduced to properly balance both the trust management and privacy-preservation scheme to improve the robustness in the vehicular networks^61^.

Such an adaptive trust management model can be enforced in the automated negotiation system for improving the bargaining process occurs among the consumers and providers, by encouraging the negotiation with the trusted participants. In this research work, the development of trusted negotiation system is identified as a key study problem that can maximize the success rate amongst the bargaining parties. An important requirement in such trusted negotiation framework is trust that allows the cloud participants to interact with confidence and establishes mutual trust which gives the higher possibility of further negotiation opportunity. Addressing this requirement, only few relevant research works were identified in the cloud computing literatures and its detailed information is given in the next section.

### Trusted negotiation frameworks

According to the cloud-based literature survey, the trusted negotiation framework can be broadly classified into three types namely peer-to-peer, mediated (broker-based) and distributed (multi-brokering) approaches. Current research study exploited an Automated Trust Negotiation (ATN) system that can create the common trust among the negotiating participants wanting to conduct business and share incomes by demanding and revealing numerally signed identifications. This framework guarantees the success rate of negotiation and defense of complex evidence by using the trust negotiation strategy in the negotiating agents^62^. A trust negotiation framework is designed for enabling the strangers to eventually collaborate and incrementally learn about each other. So, trust agents are used to automate the negotiation request that carries out a negotiation conversation between a pair of trust agents by gradually exchanging trust instances. The behavior of trust agents are defined through a set of meta-policies which drive the negotiation session governed by a negotiation protocol^63^. A decentralized (peer-to-peer) trust model contains the negotiation and enforcement process that allows the bargaining parties to evade the lying behavior of other parties. Individual consumer can examine the providers report and then informs the honesty values of the other parties. This value can be exploited to bargain the prices in the future negotiation processes^64^. A trust relation on e-negotiation adaption designates the interaction among many trust perceptions which can straight away touch the intension to involve in the goal behavior of e-commerce negotiation usage^65^. A comprehensive XML-based Trust-X negotiation framework was proposed in a peer-to-peer cyberspace setting, where sensitive interactions happen among entities without any preceding information of each other. This framework has a complementary module for managing and caching the previously used trust sequences as sequence prediction module^66^. An identity based federated trust negotiation model supports the cloud-enabled utility service for ensuring a consistent trust formation among service and identity providers^67^. Above negotiation frameworks guarantee the reliable trust negotiation among the participants and works according to the context of peer-to-peer approach. However, these frameworks and their strategies do not ensure the optimization of bargaining process amongst the cloud-based negotiation participants. Moreover, the existing negotiation frameworks suffer from the trust evaluation overhead at the negotiating end.

As per recent negotiation studies, very few investigations are offered in the context of peer-to-peer approach. Remaining mediated and distributed approaches are mostly under research investigation for future enhancement relating to reputation, functional and non-functional features^68^. This proposed research study emphases on the development of broker-based (mediated) trusted cloud service negotiation framework for overcoming the trust evaluation overhead that occurs at the negotiating peers. Moreover, the projected broker-based cloud-enabled e-commerce negotiation framework with adaptive probabilistic trust management model will improve the success rate and utility value by minimizing the bargaining conflict occurring between the participants present in cloud trading market.

## Problem formulation of adaptive probabilistic trust management model

In cloud-based setting, the behaviors of the service provider agents are uncertain or unpredictable during the real time negotiation process. To analyze the factor influencing the degree of trust worthiness and make use of adaptive probabilistic trust management model for articulating the dynamism and subjective uncertainty of trust computation factors about the negotiation participants (incomplete opponent service provider agent). The context of an adaptive probabilistic trust management model is expressed by seven-tuple $\:\langle\text{X},\:\text{Y},\:\text{F},\:\text{A},\:\text{R},\:\text{S},\:\text{H}\rangle$. Let $\:X=\left\{{ITBA}_{1},{ITBA}_{2},\dots\:,{ITBA}_{n}\right\}$ and $\:Y=\left\{{SPA}_{1},{SPA}_{2},\dots\:,{SPA}_{m}\right\}$ denote the set of intellectual third-party broker agent (trustors) and service provider agents (trustees) correspondingly. Then, the trust feature or event $\:F=\left\{{f}_{SR},{f}_{CR},{f}_{HR}\right\}$ denotes the set of trust components like success rate, cooperation rate, and honesty rate that define the trust values of collection of negotiation attribute $\:A=\left\{{a}_{1},{a}_{2},\dots\:,{a}_{n}\right\}$. Let $\:R=\left\{{r}_{1},{r}_{2},\dots\:,{r}_{k}\right\}$ signify the different trust rankings of provider agents and $\:S=\left\{{s}_{1},{s}_{2},\dots\:,{s}_{k}\right\}$ represent the quantity of negotiation states present during the bargaining process. Then, $\:H$ denotes the history or degree of bargaining knowledge acquired in the bilateral negotiation process during time period $\:T$. It provides the degree of trust worthiness about provider agents by determining the weighted trust values of evaluation attributes available in cloud based trusted negotiation framework.

## Cloud-enabled e-commerce negotiation framework

**Fig. 2:**
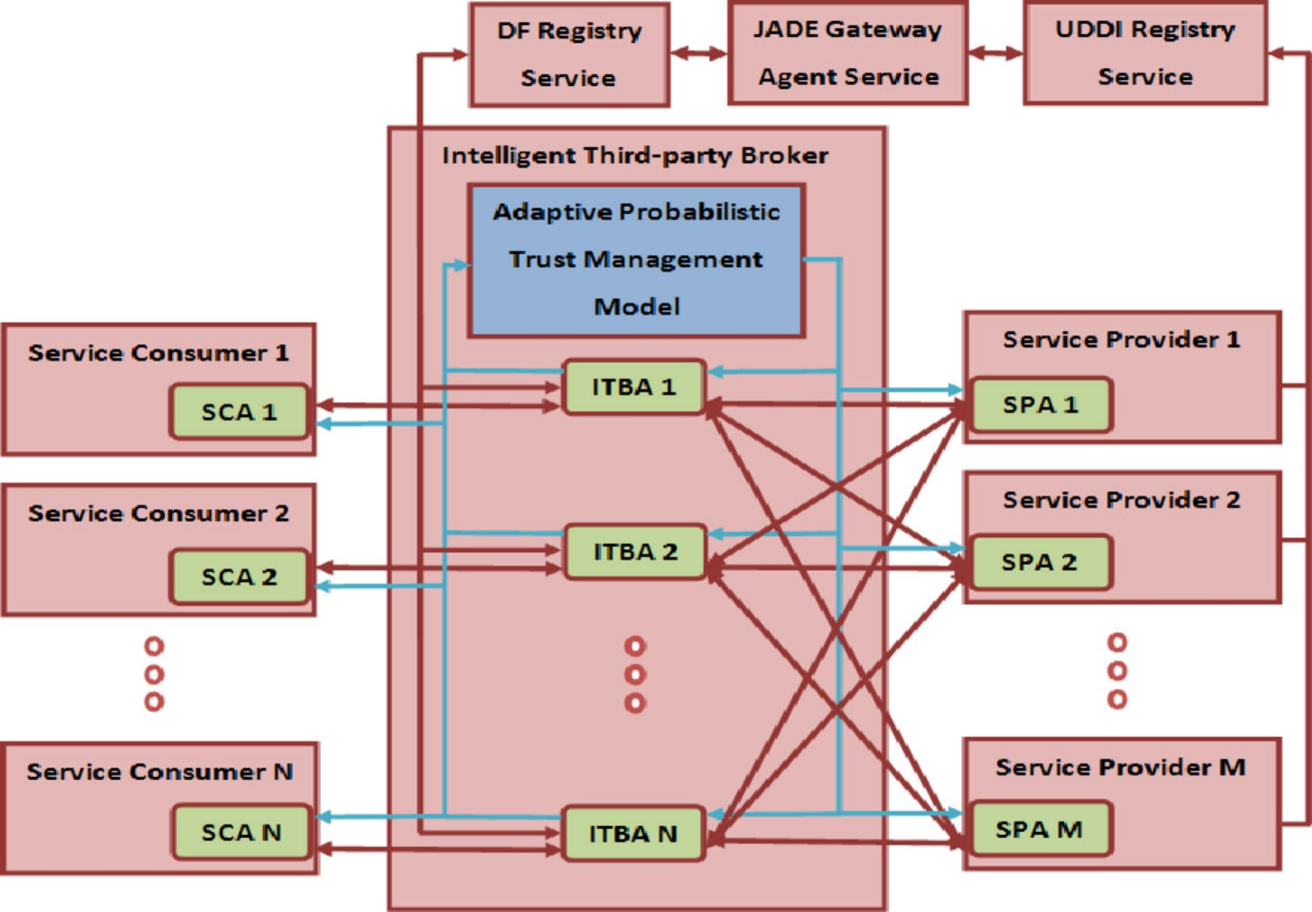
Architecture of Broker-based Cloud-enabled E-commerce Negotiation Framework.

The architecture of broker-based cloud-enabled e-commerce negotiation framework (BCENF) is proposed as exposed in Fig. 2. It is an enhanced model of ADSLANF^70^, presented in the previous research works with an emerging trust management model. This proposed framework consists of a set of service consumer agent (SCA), intelligent third-party broker agent (ITBA) and service provider agent (SPA) to negotiate the cloud service on behalf of the service consumers, broker, and provider agent correspondingly. A SCA will generate the negotiation request to an ITBA for identifying the highly trusted and best cloud service provider within the stipulated time. To negotiate the required service, ITBA lookups the directory facilitator registry for confirming the availability of services deployed in cloud trading market. This service availability information is frequently updated by the JADE entry service which reflects the service variations occurring in the Universal Description Discovery and Integration (UDDI) registry. UDDI registry contains the description of numerous cloud services published by many service providers. After identifying the service availability, the two-sided bargaining process is initiated among the ITBA and the set of SPAs. These agents can negotiate the cloud service in the aspects of price, speed, and time-slot attributes. The ITBA produces the offer according to the opponent’s counter offers and the trust decision suggested by the Bayesian based adaptive probabilistic trust management model. It generates the provider trust value according to the Bayesian-based trust evaluation modeling enforced in the ITBA.

### Bayesian-based adaptive probabilistic trust management model

**Fig. 3:**
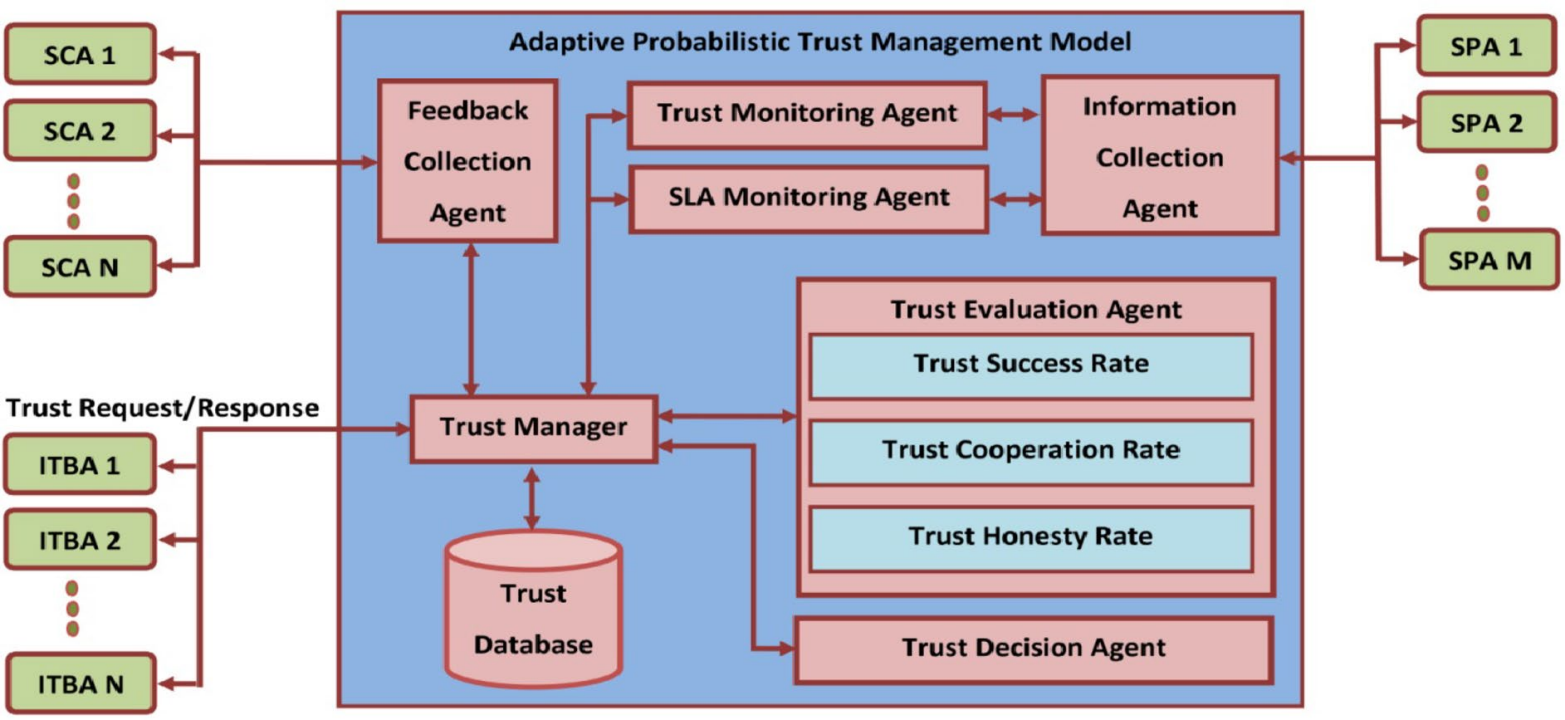
Architecture of Bayesian-based Adaptive Probabilistic Trust Management Model.

An abstract architecture of the expected Bayesian-based Adaptive Probabilistic Trust Management Model is designed as shown in Fig. 3. It includes several internal components like trust manager, trust database, feedback collection agent, trust monitoring agent, SLA monitoring agent, information collection agent, trust evaluation agent, and trust decision agent. The trust manger component is invoked by the ITBA for generating valuable offer to the negotiating participant, according to the counter offer and available trust evidence about the SPAs. This trust evidence is collected from the trust database component which contains the belief history of all SPAs involved in past negotiation processes. According to the trust values available in the trust database, the trust monitoring agent will periodically monitor and update trust values of all service provider agents by gathering information from the information collection agent. To identify the un-trusted service providers, an SLA monitoring agent is exploited to monitor the behavior and concert of all the agreed service provisioned by the various service provider agents. This agent in turn frequently invokes the information collection agent for evaluating trust value of the entire SPA over the agreed service. To further strengthen the evaluated trust values, a feedback collection agent is used to collects the subjective feedback from various service consumer agents who have already interacted and committed with such service provider agent. This feedback collection is used for evaluating the correctness of the previously suggested trust information about various service provider agents.

After monitoring the trust and SLA, the trust manager collects the feedback to assess the trust value and decision regarding the opponent’s offer requested by the corresponding broker agent. Therefore, the trust evaluation agent and trust decision agent are invoked by the trust manager at each stage of negotiation process for suggesting effective behavioral decision to the broker agent. Initially, the trust evaluation agent will assess the trust value of the SPAs based on the interaction, compliance, and recommendations to check whether the performance of negotiated service is working as per the agreed SLAs of cloud negotiation participants. The above sequence of trust factors evaluation and decision during the negotiation process is generalized into Bayesian based Adaptive Probabilistic Trust Management Modeling pseudo-code as shown in Algorithm 1. Based on the evaluation, trust decision agent is invoked to take the appropriate behavioral decisions according to the policy and negotiation model. So, the Bayesian based adaptive probabilistic trust management modeling is proposed for formulating behavioral decisions among the negotiation participants involved in the multi-agent broker-based cloud-enabled e-commerce negotiation framework.

**Algorithm 1:**
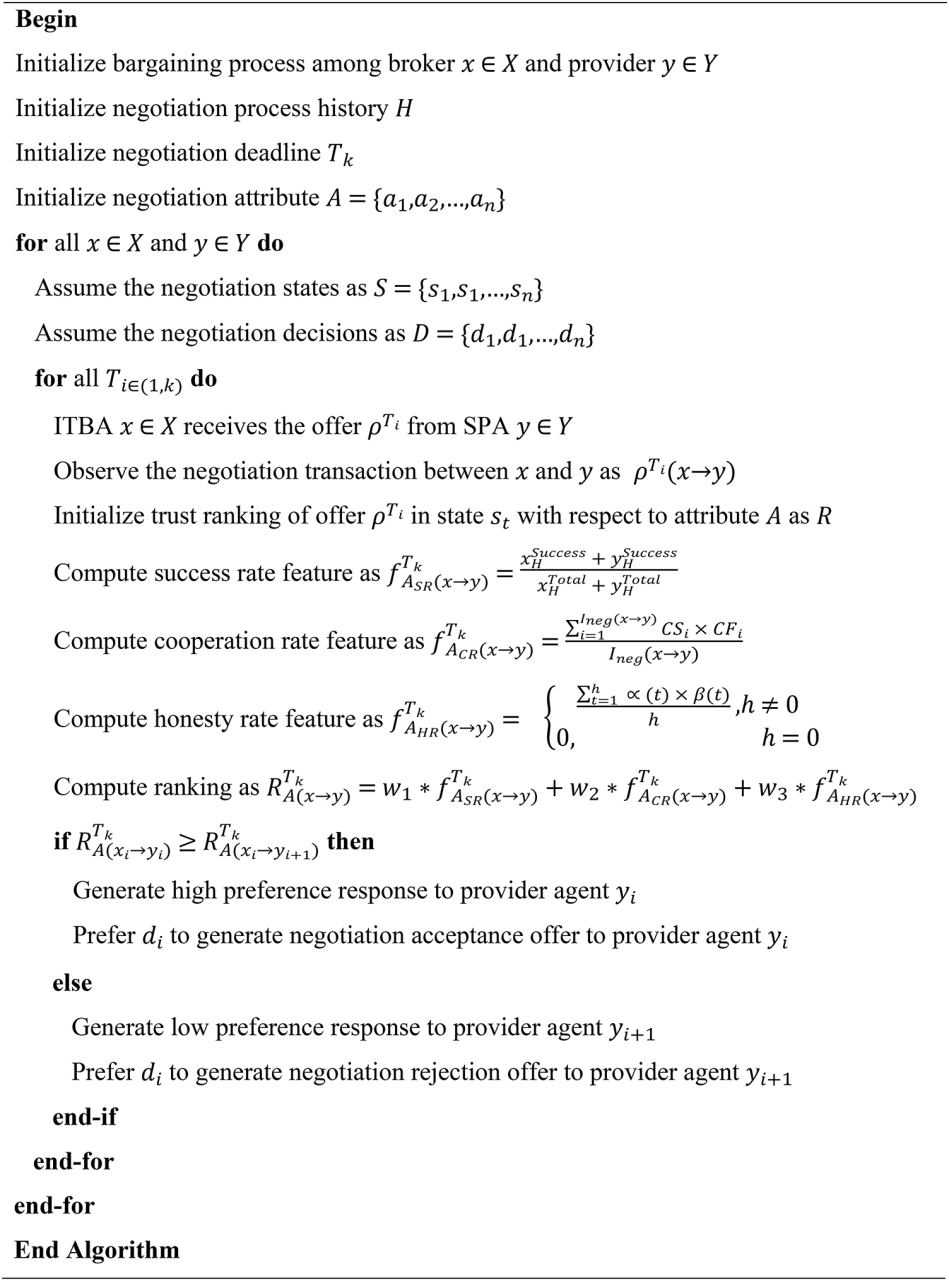
Adaptive probabilistic trust management modeling.

### Modeling of Bayesian-based adaptive probabilistic trust management model

Bayesian-based likelihood compactness function is used to represent the distribution of trust values (worthiness) of negotiating participant’s transaction according to negotiation history. A negotiation transaction $\:\rho\:$ happens when the service consumer agent accepts the negotiated service from the SPA. Assume the ITBA initiates the negotiation process with a service provider agent based on the negotiation request received from service consumer agent. In the real time multi-cloud setting, the SPAs are ranked by the SPA as sequence of integers $\:(\text{1,2},\text{3,4},5)$ for initiating the actual negotiation process. Here, the value 1 to 5 denotes lowest, low, medium, high, and highest ranks correspondingly. The negotiation transaction $\:\rho\:(x\to\:y)$ denotes the transaction between broker agent (on behalf of consumer agent) $\:x$ and provider agent $\:y$ associated with feature vector $\:F=\left\{{f}_{SR},{f}_{CR},{f}_{HR}\right\}$. To effectively combine various features, the values of each feature is normalized to the same range, i.e., $\:\left[\text{0,1}\right]$. Further, the ranking is analyzed accordingly by the normalized feature value $\:{r}_{i}\in\:\left[\text{0,1}\right]$ that could be segregated as $\:k$ equally restricted ranks $\:({r}_{1},\:{r}_{2},\:\dots\:,\:{r}_{k})$ such as ($\:0\le\:{r}_{i}\le\:1$). Therefore, the ranking that follows a uniform probability distribution over the k-dimensional rating space is called as continuous distribution function indexed by the multiple parameters. Let $\:{p}_{i}=P\left({r}_{i}\right)$ be the probability of the service provider agent for obtaining the ranking $\:{r}_{i}$, such that $\:\sum\:_{i=1}^{k}{p}_{i}=1$. Let $\:{\mathcal{S}}_{i}$ be the number of ranking occurrences $\:{r}_{i}$ over the ranking sample $\:\mathcal{S}$ and it can be defined as $\:n=\sum\:_{i=1}^{k}{\mathcal{S}}_{i}$.

The primary goal of using Bayesian learning approach in adaptive probabilistic trust management model is to summarize the distribution of trust ranking of service provider agent through the conditional probability density function such as prior and posterior distribution. Prior distribution collects the subjective information about the trust values obtained prior to the ranking sample $\:\mathcal{S}=({\mathcal{S}}_{1},\:{\mathcal{S}}_{2},\dots\:,{\mathcal{S}}_{k})$. After obtaining $\:\mathcal{S}$, the prior distribution can be updated to posterior distribution. To predict the behavior of service provider agents at every phase of bargaining process, the Bayesian-based adaptive probabilistic trust management model makes use of prior probability of the negotiation trust feature and later updates it in the available trust database evidence. Due to lack of additional information about the opponent, it first assumes the prior distribution $\:f\left(V\right)$ as a uniform distribution and then estimates the posterior distribution according to ranking sample $\:\mathcal{S}=({\mathcal{S}}_{1},\:{\mathcal{S}}_{2},\dots\:,{\mathcal{S}}_{k})$ that conforms to the multinomial distribution as shown in Eqs. (1) and (2) respectively.

1 $$\:f\left(\mathcal{S}\right|V)=\frac{n!}{{\varPi\:}_{i=1}^{k}\left({\mathcal{S}}_{i}!\right)}{\varPi\:}_{i=1}^{k}({{p}_{i}}^{{\mathcal{S}}_{i}})\:$$

2 $$\:f\left(V\right|\mathcal{S})=\frac{f\left(\mathcal{S}|V\right)\times\:f\left(V\right)}{\underset{0}{\overset{1}{\int\:}}{\int\:}_{0}^{1}\dots\:{\int\:}_{0}^{1}f\left(\mathcal{S}|V\right)\times\:f\left(V\right)\times\:d{p}_{1}\times\:d{p}_{2}\times\:\dots\:d{p}_{k-1}}\:$$

Let $\:V=({p}_{1},{p}_{2},\dots\:,{p}_{k-1})$ and $\:{p}_{k}=1-\sum\:_{i=1}^{k-1}{p}_{i}$. After substituting the vales in Eq. (2), it can be rewritten as shown in Eq. (3).

3 $$\:f\left(V\right|\mathcal{S})=\frac{{(1-\sum\:_{i=1}^{k-1}{p}_{i})}^{{\mathcal{S}}_{k}}\times\:{\varPi\:}_{i=1}^{k-1}\left({{p}_{i}}^{{\mathcal{S}}_{i}}\right)}{\underset{0}{\overset{1}{\int\:}}{\int\:}_{0}^{1}\dots\:{\int\:}_{0}^{1}{(1-\sum\:_{i=1}^{k-1}{p}_{i})}^{{\mathcal{S}}_{k}}\times\:{\varPi\:}_{i=1}^{k-1}\left({{p}_{i}}^{{\mathcal{S}}_{i}}\right)\times\:d{p}_{1}\times\:d{p}_{2}\times\:\dots\:d{p}_{k-1}}\:$$

Further, the trust value under certainty can be measured according to the statistical measurement that states the cumulative probability distribution of $\:V$ must be 1 within $\:\varOmega\:$. It follows the function $\:g:\varOmega\:=\left[\text{0,1}\right]\times\:\left[\text{0,1}\right]\times\:\dots\:\times\:\left[\text{0,1}\right]\to\:[0,\infty\:)$, that gives an integral value of uniform distribution $\:g\left(V\right)$ as $\:{\int\:}_{0}^{\infty\:}g\left(V\right)\times\:dV=1$ and mean value within $\:\varOmega\:$ as $\:\frac{{\int\:}_{0}^{\infty\:}g\left(V\right)\times\:dV}{{(1-0)}^{k-1}}=1$. Therefore, the ranking sample under certainty can be defined as shown in Eq. (4).

4 $$\:{{r}_{i}}^{certainty}=\frac{1}{2}\:\:{\int\:}_{0}^{\infty\:}\left|\frac{{(1-\sum\:_{i=1}^{k-1}{p}_{i})}^{{\mathcal{S}}_{k}}\times\:{\varPi\:}_{i=1}^{k-1}\left({{p}_{i}}^{{\mathcal{S}}_{i}}\right)}{{\int\:}_{0}^{\infty\:}({(1-\sum\:_{i=1}^{k-1}{p}_{i})}^{{\mathcal{S}}_{k}}\times\:{\varPi\:}_{i=1}^{k-1}({{p}_{i}}^{{\mathcal{S}}_{i}}\left)\right)\times\:dV}-1\right|\times\:dV\:$$

Let $\:\frac{1}{2}$ be the midpoint of ranking range $\:\left[\text{0,1}\right]$ represent the neutral belief between distrust $\:[0,\:\frac{1}{2})$ and trust $\:(\frac{1}{2},0]$ that can be projected as negative and positive ranking respectively.

The proposed Bayesian based adaptive probabilistic trust management model is more suitable under uncertain behavioral information about the opponent. Therefore, broker agent considers the degree of success rate $\:{SR}^{T}$, cumulative cooperation rate $\:{CR}^{T}$ and honest rate $\:{HR}^{T}$ as three trust feature of opponent’s negotiation attribute during time $\:T$. At any time $\:{T}_{k}$, a trust rate ranking of the attribute $\:A$ over the negotiation state $\:{S}_{k}$ is assumed to be $\:{R}_{A}^{{T}_{k}}$. Then, the trust rate at period $\:{T}_{k+1}$ can be computed as shown in Eq. (5), by using the previous state trust value ranking.

5 $$\:{R}_{A}^{{T}_{k+1}}=\left(\frac{1-\theta\:}{{R}_{A(x\to\:y)}^{{T}_{k}}}+\frac{\theta\:}{{R}_{A(x\to\:y)}^{{T}_{k+1}}}\right)$$

Where the parameter $\:\theta\:\in\:\left(\text{0,1}\right)$ denotes the nearest integer function that controls the decay of previous state trust value and the contribution of new negotiation state trust value. In case of getting a high trust value at the lateral negotiation state, the global trust rate of the attribute will have only small significant changes. Similarly, the global trust rate of the attribute will fall sharply due to low trust value at the lateral negotiation state. Here, the symbol $\:A(x\to\:y)$ denotes the trust representation of service provider agent $\:y\in\:Y$ by the trustee or intelligent third-party broker agent $\:x\in\:X$ over the attribute $\:A$. This peer-to-peer trust representation of attribute considers the three equally weighted trust components as shown in Eq. (6).

6 $$\:{R}_{A(x\to\:y)}^{{T}_{k}}={w}_{1}*{f}_{{A}_{SR}(x\to\:y)}^{{T}_{k}}+{{w}_{2}*f}_{{A}_{CR}(x\to\:y)}^{{T}_{k}}+{{w}_{3}*f}_{{A}_{HR}(x\to\:y)}^{{T}_{k}}\:$$

Let $\:{w}_{1}$, $\:{w}_{2}$ and $\:{w}_{3}$ are the weights associated with the respective trust features like success rate, cooperation rate and honesty rate with $\:{w}_{1}+{w}_{2}+{w}_{3}=1$.

The success rate between the negotiation participants can be defined as shown in Eq. (7). It characterizes the participants range of acceptance during the bargaining history $\:\text{H}$.

7 $$\:{f}_{{A}_{SR}(x\to\:y)}^{{T}_{k}}=\frac{{x}_{H}^{Success}+{y}_{H}^{Success}}{{x}_{H}^{Total}+{y}_{H}^{Total}}\:\:\:$$

Where $\:{x}_{H}^{Success}$ and $\:{y}_{H}^{Success}$ represent the number of consensuses reached by the agent $\:x$ and $\:y$ respectively during negotiation history $\:H$. Let $\:{x}_{H}^{Total}$ and $\:{y}_{H}^{Total}$ denote the total number of negotiation transaction attempted by the respective agent $\:x$ and $\:y$. A cooperation rate $\:{f}_{{A}_{CR}(x\to\:y)}^{{T}_{k}}$ of the opponent provider agent can be defined by the broker agent based on the credible satisfaction received from each negotiation transaction that occurs during the time $\:{T}_{k}$. It can be computed by Eq. (8).

8 $$\:{f}_{{A}_{CR}(x\to\:y)}^{{T}_{k}}=\frac{\sum\:_{i=1}^{{I}_{neg}(x\to\:y)}{CS}_{i}\times\:{CF}_{i}}{{I}_{neg}(x\to\:y)}\:\:\:$$

Let $\:{I}_{neg}(x\to\:y)$ denote the total amount of negotiation interactions (communications) that occurs among the participants, $\:{CS}_{i}$ denote the normalized amount of credible satisfaction received during the negotiation process and $\:{CF}_{i}$ represent the normalized amount of credible feedback that tells the evidence of the service quality offered by the opponent.

The honesty rate trust factor between $\:x\in\:X$ and $\:y\in\:Y$ can be defined according to history of negotiation process as shown in Eq. (9), that assumes the negotiation satisfaction degree of $\:y$ as a sequence of probabilistic rating $\:\propto\:\left(1\right),\propto\:\left(2\right),\dots\:,\propto\:\left(h\right)$, say $\:t\in\:[1,h]$ and $\:0\le\:\propto\:\left(t\right)\le\:1$.

9 $$\:{f}_{{A}_{HR}(x\to\:y)}^{{T}_{k}}=\:\:\:\left\{\begin{array}{c}\frac{\sum\:_{t=1}^{h}\propto\:\left(t\right)\times\:\beta\:\left(t\right)}{h},h\ne\:0\\\:0,\:\:\:\:\:\:\:\:\:\:\:\:\:\:\:\:\:\:\:\:\:\:\:\:\:\:\:\:\:\:\:\:\:h=0\end{array}\right.\:\:$$

Where $\:\beta\:\left(t\right)$ represents an attenuation function that can be used to weigh the historic experience over the time stamp t as defined in Eq. (10).

10 $$\:\left\{\begin{array}{c}\beta\:\left(t\right)=1,\:\:\:\:\:\:\:\:\:\:\:\:\:\:\:\:\:\:\:\:\:\:\:\:\:\:\:\:\:\:\:\:\:\:\:\:\:\:\:\:\:\:\:t\ne\:h\\\:\beta\:\left(t-1\right)=\beta\:\left(t\right)-{(1-\mu\:)}^{h},\:\:\:\:\:\:t=h\end{array}\right.\:\:\:$$

Let the adjustable positive constant $\:\mu\:$ and $\:h$ denote the historic evidences. It is clear from the estimation of honesty rate trust factor; the trust value will increase only with respect to the reflection of positive experiences about the negotiation opponent’s $\:y$ over the time period $\:t$.

The performance assessment parameters such as total negotiation time, communication overhead, utility value, and success rate are considered in this research study. The total negotiation time $\:{\tau\:}_{TNT}$ can be computed as defined in Eq. (11).

11 $$\:{\tau}_{TNT}={\mathcal{D}}_{SCA}+{\tau}_{CT(SCA,ITBA)}+\left(k\times\:\sum\:_{i=1}^{m}\left({\mathcal{D}}_{ITBA}+{\tau}_{LT}\right)\right)+{\tau}_{CT(ITBA,\:SPA)}+{\mathcal{D}}_{SPA}\:$$

Where the value $\:{\mathcal{D}}_{SCA}$, $\:{\mathcal{D}}_{ITBA}$, and $\:{\mathcal{D}}_{SPA}$ denotes the expected service negotiation delay time of $\:SCA$, $\:ITBA$, and $\:SPA$ respectively. The $\:{\tau\:}_{CT(SCA,ITBA)}$ represents the communication time taken between the $\:SCA$ and $\:ITBA$, $\:{\tau\:}_{LT}$ represents the service lookup time, and $\:{\tau\:}_{CT(ITBA,\:SPA)}$ represents the communication time taken between the $\:ITBA$ and $\:SPA$. Finally, k denotes the number of concurrent negotiations initiated by the $\:ITBA$ with respect to multiple SPAs. Next, the communication overhead $\:{CO}_{x\leftrightarrow\:y}$ involves in the negotiation process can be estimated as shown in the Eq. (12).

12 $$\:{CO}_{x\leftrightarrow\:y}={\mathcal{I}}_{SCA\leftrightarrow\:ITBA}+{\mathcal{I}}_{ITBA\leftrightarrow\:SPA}\:$$

The total utility value of the offer $\:{\rho\:}_{x}$ and counter-offer $\:{\rho\:}_{y}$ can be computed as expressed in the Eqs. (13) and (14) respectively.

13 $$\:{\mathcal{U}}^{Total}\left({\rho\:}_{x}\right)=\sum\:_{i=1}^{n}\mathcal{W}\left({\mathcal{X}}_{i}\right)\times\:{\mathcal{U}}_{{\rho\:}_{x}}\left({\mathcal{X}}_{i}\right)\:$$

14 $$\:{\mathcal{U}}^{Total}\left({\rho\:}_{y}\right)=\sum\:_{i=1}^{n}\mathcal{W}\left({\mathcal{X}}_{i}\right)\times\:{\mathcal{U}}_{{\rho\:}_{y}}\left({\mathcal{X}}_{i}\right)\:$$

Let $\:\mathcal{W}\left({\mathcal{X}}_{i}\right)$ be the weight of negotiation attributes $\:\left({\mathcal{X}}_{1},{\mathcal{X}}_{2},\dots\:,{\mathcal{X}}_{n}\right)$ used in the offer or counter-offer which ranges from 0 to 1, $\:{\mathcal{U}}_{{\rho\:}_{x}}\left({\mathcal{X}}_{i}\right)$ be the utility value of the $\:SCA$ participant $\:x$ with respect to negotiation offer $\:{\rho\:}_{x}$, and $\:{\mathcal{U}}_{{\rho\:}_{y}}\left({\mathcal{X}}_{i}\right)$ be the utility of the $\:SPA$ participant $\:y$ with respect to negotiation offer $\:{\rho\:}_{y}$. Here, the utility value ranges from 0 to 1, where 1 represents the full satisfaction and 0 represents the dissatisfaction of the negotiation participants. Finally, the success rate between the participants $\:x$ and $\:y$ can be computed by using the Eq. (15).

15 $$\:\mathcal{S}\mathcal{R}=\frac{{{x}_{i}}^{Success}+{{y}_{i}}^{Success}}{{{x}_{i}}^{Total}+{{y}_{i}}^{Total}}\:\:$$

Where $\:{{x}_{i}}^{Success}$ and $\:{{y}_{i}}^{Success}$ represents the number of negotiation participants from $\:{x}_{i}$ and $\:{y}_{i}$ who reaches the negotiation agreement. Similarly, the value $\:{{x}_{i}}^{Total}$ and $\:{{y}_{i}}^{Total}$ represents the number of participants involved in the negotiation process from $\:{x}_{i}$ and $\:{y}_{i}$ respectively.

## Experimental evaluation

Experimental setting is created through the JADE simulation tool^69^ that imitates the real-time negotiation scenario of cloud participants bargaining in the multi-cloud setting. This simulation considers the performance evaluation parameters like success rate and utility value of participants as defined in previous research work^70^. Here, the existing frameworks like negotiation framework architecture (NFA), cloud negotiation model (CNM) and automated dynamic SLA negotiation framework (ADSLANF) explored in the previous research study were used for the comparison of results with the proposed BCENF. The key reason behind choosing these existing negotiation frameworks is due to the exploitation of proper negotiation protocol mechanism in their experimentation. This protocol only gives the clear picture of negotiations happening between the negotiating parties with appropriate negotiation tactics and decision-making process. To start the simulation, the negotiation attributes like price and time slots are initialized on both sides of negotiation participants for initiating the real bargaining process between the negotiation participants. The front-end negotiation portal of ITBA and SPA that initialize the bargaining attributes are very much similar like the diagrams shown in our previous research study. The input value of all the negotiation framework algorithm includes the input attributes (such as Initial Price, Reserved Price, Initial Time-slot, Reserved Time-slot, Negotiation Deadline, Negotiation Agent) given in the Table 1. Firstly, the initial output parameters observed over the negotiation parameters are given in the Table 2. Here, the service negotiation delay $\:{\mathcal{D}}_{SCA}$, $\:{\mathcal{D}}_{ITBA}$, and $\:{\mathcal{D}}_{SPA}$ observed in the existing NFA, CNM, and ADSLANF frameworks are 10 s, whereas the proposed framework takes only 3 s. Similarly, the communication time $\:{\tau\:}_{CT(SCA,ITBA)}$ taken between the $\:SCA$ and $\:ITBA$ is 10 s, and the communication time $\:{\tau\:}_{CT(ITBA,\:SPA)}$ taken between the $\:ITBA$ and $\:SPA$ is 5 s. In addition, the service lookup time $\:{\tau\:}_{LT}$ is 10 s. Consequently, the final output parameter with varying values of utility value and success rate are observed with respect to various negotiation rounds (such as 50, 100, 200, and 500 rounds) as depicted in Table 3.

**Fig. 4:**
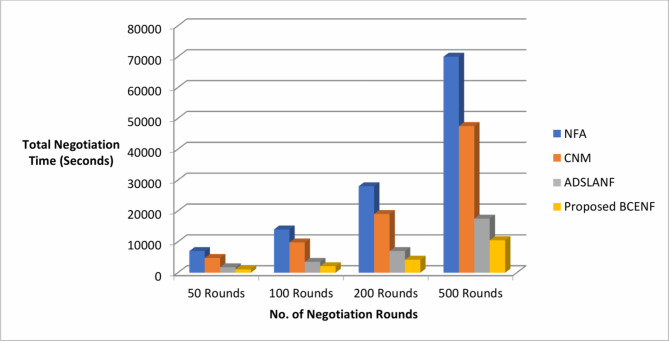
Negotiation performance with respect to total negotiation time.

**Fig. 5:**
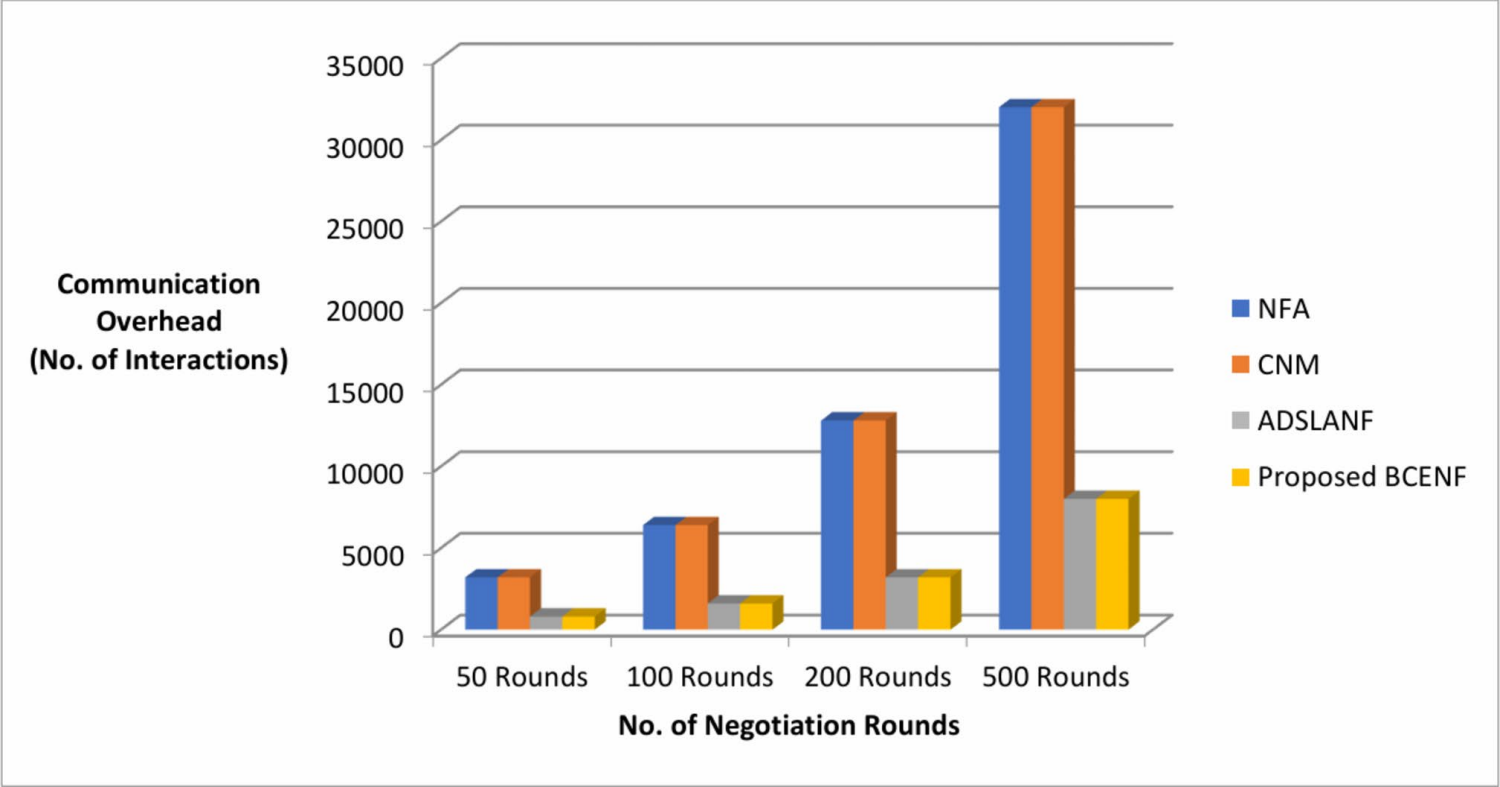
Negotiation performance with respect to communication overhead.

Next, a sniffer agent is introduced in the framework to visualize and monitor the two-sided bargaining process between the participants. This research work considers the benchmark dataset of previous research work as shown in Table 1 to initialize the negotiation attributes at the front-end negotiation portal of ITBA and SPAs. After starting the simulation of bargaining process, the results of various negotiation process with respect to total negotiation time, and communication overhead are observed as reflected in the Table 2. The comparative analysis of proposed BCENF with the existing NFA, CNM and ADSLANF models are is illustrated in terms of total negotiation time and communication overhead as shown in Figs. 4 and 5.

**Table 1:** Experimental setting of broker and provider agents.

Input Attributes	Settings	
	Broker Agent	Provider Agent
Initial Price	[10,60]	[200,250]
Reserved Price	[200,250]	[10,60]
Initial Time-slot	[10,60]	[300,350]
Reserved Time-slot	[300,350]	[10,60]
Negotiation Deadline	[50,200] Rounds	[50,200] Rounds
Negotiation Agent	[5,20]	[5,20]

**Table 2:** Performance of negotiation frameworks with respect to 4 × 4 participants.

Various Frameworks	Total Negotiation Time (Seconds)				Communication Overhead (No. of Interactions)			
	50 Rounds	100 Rounds	200 Rounds	500 Rounds	50 Rounds	100 Rounds	200 Rounds	500 Rounds
NFA	7015	14,015	28,015	70,015	3202	6402	12,802	32,002
CNM	4765	9815	19,015	47,515	3202	6402	12,802	32,002
ADSLANF	1765	3515	7015	17,515	802	1602	3202	8002
BCENF	1058	2108	4208	10,508	802	1602	3202	8002

**Fig. 6:**
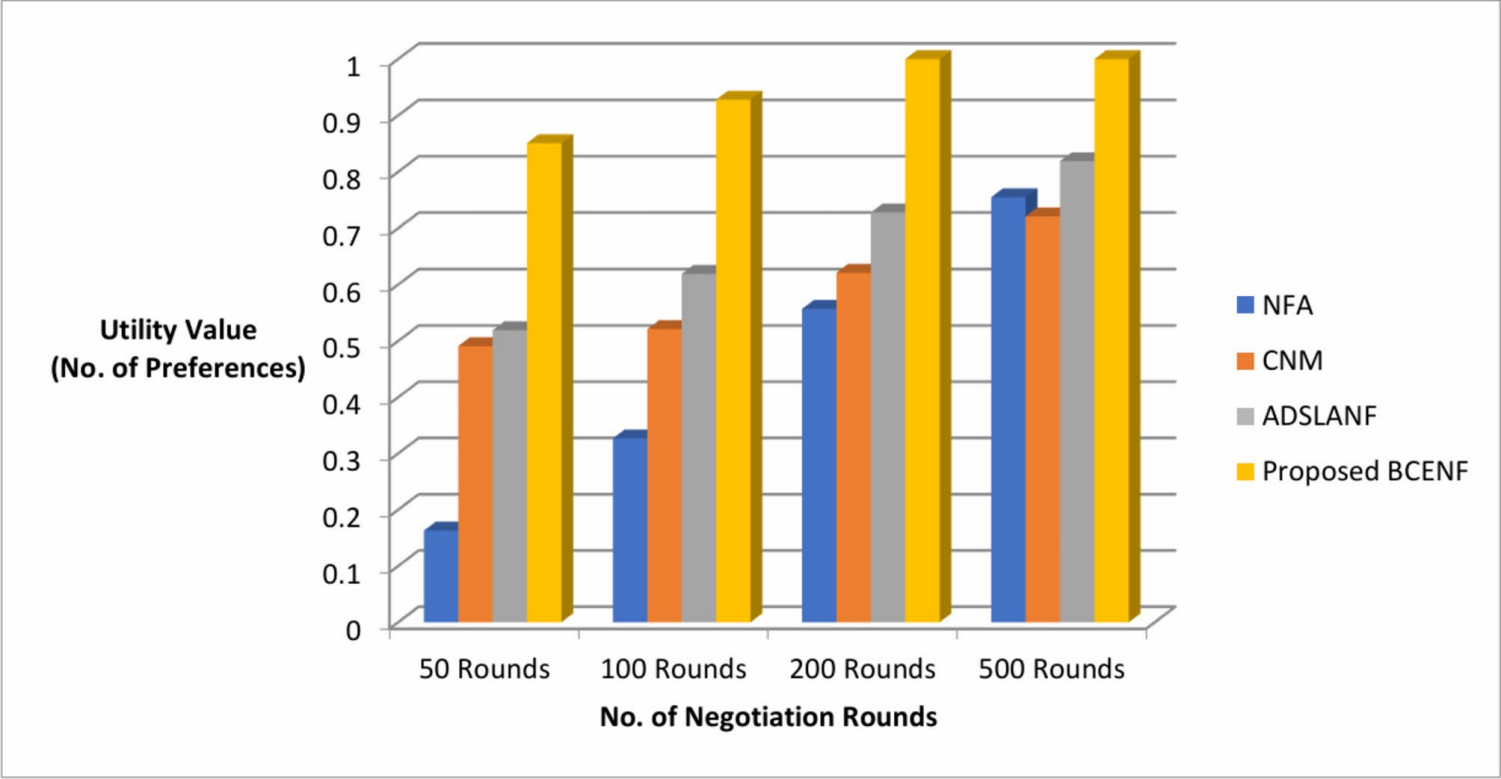
Comparison of negotiation frameworks in the aspects of utility value.

**Fig. 7:**
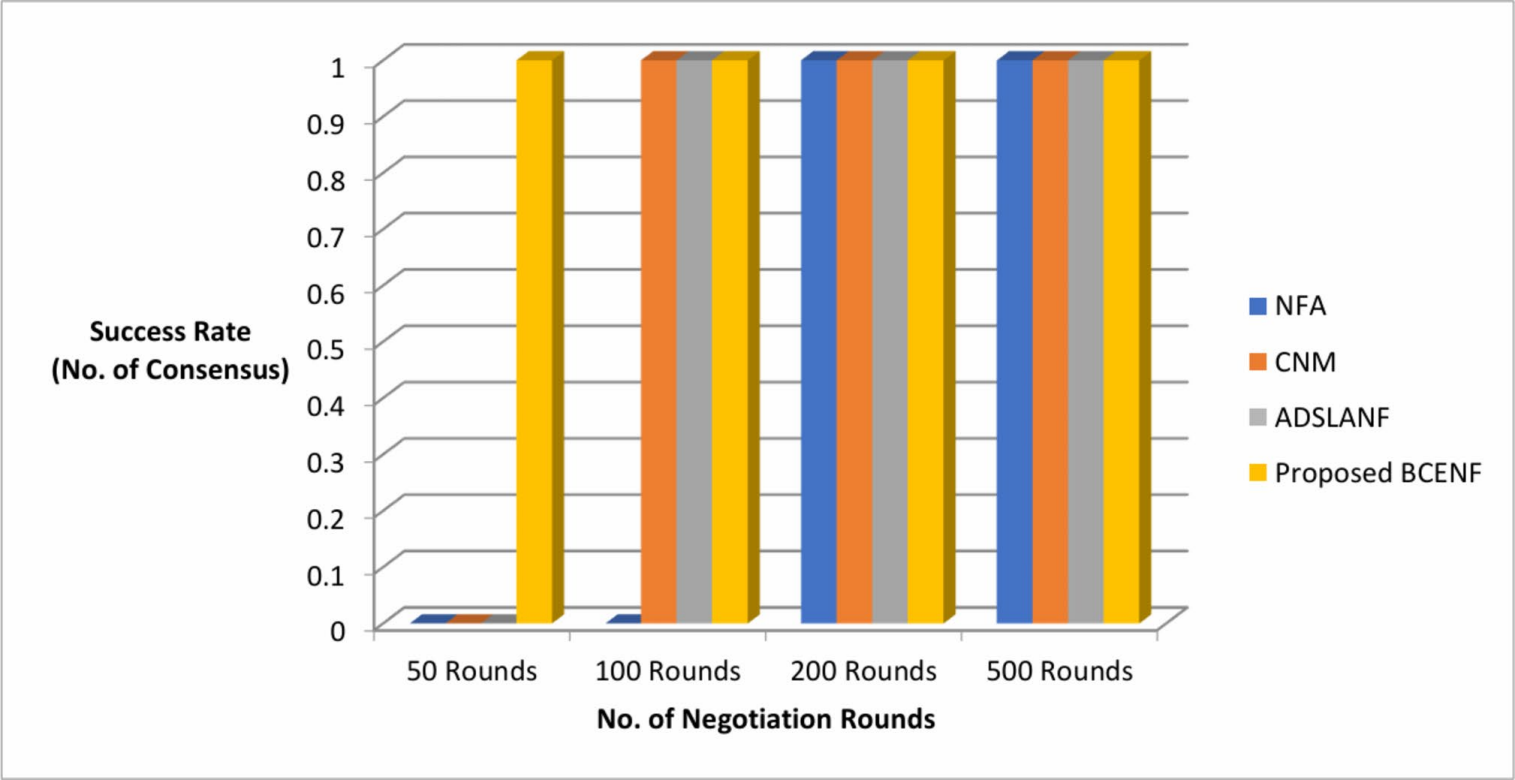
Comparison of negotiation frameworks in the aspects of success rate.

Similarly, the results of negotiation framework utility value (No. of preferences) and success rate (No. of consensus) are observed in the aspects of various bargaining rounds as depicted in Table 3. The comparison results of proposed BCENF with these existing NFA, CNM and ADSLANF models are illustrated in Figs. 6 and 7.

**Table 3:** Results of negotiation frameworks in the aspects of negotiation rounds.

Various Frameworks	50 Rounds		100 Rounds		200 Rounds		500 Rounds	
	Utility Value	Success Rate	Utility Value	Success Rate	Utility Value	Success Rate	Utility Value	Success Rate
NFA	0.163	0	0.327	0	0.557	1	0.755	1
CNM	0.491	0	0.521	1	0.621	1	0.721	1
ADSLANF	0.519	0	0.619	1	0.728	1	0.819	1
Proposed BCENF	0.851	1	0.928	1	1	1	1	1

As per the evidence of Tables 2 and 3 observations, the results of projected cloud-enabled e-commerce negotiation framework (with 50 negotiation rounds) outperforms the existing negotiation frameworks in the aspects of total negotiation time, communication overhead, utility value, and success rate. Similarly, the gradual increase in the negotiation rounds (such as 100, 200 and 500) drastically improves the projected cloud-enabled e-commerce negotiation framework performance due to the enforcement of a Bayesian-based adaptive probabilistic trust management model in the broker part. Since, this trust model identifies the trusted SPA during the negotiation process; it will improve the success rate and utility value between the participants. Moreover, the projected model not only evaluates the trustworthiness of service provider agent from the negotiation history but also progresses through the sequences of past negotiation states information.

The major importance of the proposed Bayesian-based adaptive probabilistic trust management model in the cloud-enabled e-commerce negotiation framework is the appropriate exploitation of probabilistic decision which is dynamically adapted by the broker agent. This type of dynamic decision based on trust related weight factors such as success rate, cooperation rate and honesty rate assigned to broker agent according to past negotiation state information of negotiating participant incomplete. The existing models gradually improves the success rate and utility value when there is increase in the number of bargaining rounds ranges from 200 to 500. In case of proposed model even with a smaller range of bargaining rounds, consistently delivers the maximum success rate and utility value compared to existing models due to its Bayesian based probabilistic decision taken by the broker agent. There are few limitations present in the current research study that includes the modification of negotiation protocol and workflow management among the negotiation agents that leads to communication overhead among the complex concurrent negotiation process. Further, the proposed research study can be enhanced with the machine learning based approaches to support the future trends of AI-commerce related applications.

## Conclusion and future enhancements

Proposed research study summarizes the operations of existing negotiation frameworks and establishes a broker-based cloud-enabled e-commerce negotiation framework for improving the success rate and utility value between the participants in multi-cloud environment. Here, the ITBA generates the sequence of offers based on counter offers and trust worthiness of the provider agent. The degree of trust worthiness is measured by the proposed Bayesian based adaptive probabilistic trust management model that has more reasonable and practical significance when compared to the existing trust management models. Similarly, the proposed trust management model leverages the negotiation process of ITBAs without any conflict with the SPAs. The validity of the proposed broker-based cloud-enabled e-commerce negotiation framework is demonstrated through exhaustive simulation experiments which shows significant improvement over the existing negotiation frameworks. Further improvements can be made in this research study by exploring the cognitive fuzzy learning mechanisms to drastically improve the success rate and utility value without causing any bargaining conflicts between the negotiation participants.

## Data Availability

The datasets used and/or analysed during the current study available from the corresponding author on reasonable request.
